# Comparative Analysis of Taste Perception Among Airline Pilots, Construction Workers, and Office Employees

**DOI:** 10.7759/cureus.69361

**Published:** 2024-09-13

**Authors:** Piercarlo Minoretti, Giovanni Fortuna, Davide D’Acquino, Konstantinos Lavdas

**Affiliations:** 1 Occupational Health, Studio Minoretti, Oggiono, ITA

**Keywords:** airline pilots, construction workers, occupational exposure, salivary ph, taste perception

## Abstract

Objective

Occupational exposures may influence gustatory sensations through mechanisms such as fatigue, acute or chronic stress, circadian rhythm disruptions, and exposure to various chemicals. In this cross-sectional study, we sought to compare taste perception across three professional groups, namely airline pilots, construction workers, and office employees, by assessing taste identification times for sweet, salty, sour, and bitter flavors, alongside salivary pH levels.

Methods

The study cohort consisted of 90 healthy male participants, with 30 individuals in each occupational group, matched for age and professional experience. Salivary pH was measured using pH meter paper, whereas taste identification times were assessed using aqueous solutions applied to dissolvable strips for each taste.

Results

There were no significant differences in salivary pH among the study groups. However, airline pilots exhibited a significantly longer identification time for sweet taste (9.8 ± 3.9 seconds) compared to construction workers (7.0 ± 3.1 seconds, P < 0.05) and office employees (7.1 ± 3.3 seconds, P < 0.05). Conversely, construction workers demonstrated a significantly prolonged identification time for sour taste (6.1 ± 2.9 seconds) compared to pilots (4.2 ± 2.6 seconds, P < 0.05) and office employees (4.6 ± 2.5 seconds, P < 0.05). No significant differences were observed in the identification times for salty and bitter tastes across the groups.

Conclusion

We found significant differences in taste perception among airline pilots, construction workers, and office employees, particularly concerning sweet and sour tastes. These findings suggest that occupational factors may influence gustatory function in a complex manner. Further research is warranted to explore the underlying mechanisms and potential implications for dietary habits and health within specific occupational groups.

## Introduction

Taste perception, a multifaceted sensory process, is essential for human nutrition, food preferences, and overall quality of life [1-3]. Extensive research has explored the impact of aging [4,5], medical conditions [6,7], and lifestyle factors [8,9] on gustatory function. However, the influence of occupational factors on taste perception remains an underinvestigated area. Occupational exposures can potentially affect the gustatory sensations through various mechanisms, including acute [10] or chronic stress [11], alterations in circadian rhythms [12,13], and exposure to diverse chemical compounds [14,15]. Accordingly, distinct occupational groups, such as airline pilots, construction workers, and office employees, experience different environmental exposures and working conditions that may potentially affect their sensory functions. For example, airline pilots frequently experience circadian disruptions, fatigue, a sedentary lifestyle, and numerous stressors [16], all of which have been associated with taste perception in the general population. Construction workers face physical overexertion and exposure to various hazards, including extreme temperatures, chemical and biological agents such as silica dust, organic solvents, paints, welding fumes, and sewer gases [17,18]. These factors can interact with taste receptors and affect gustation [14,15]. Conversely, office employees, although not exposed to extreme environmental conditions, may endure prolonged exposure to indoor air pollutants [19], which may lead to sensory irritation [20].

While the specific effects of occupational exposures on taste perception across different professions have not been comprehensively investigated, understanding potential differences in taste perception among these occupational groups is crucial for two reasons. Firstly, altered taste perception can significantly impact dietary habits and, in turn, nutritional status and overall health [21]. Secondly, changes in taste perception may potentially serve as indicators of harmful workplace exposures [15]. Based on these premises, this comparative study sought to investigate taste perception among three distinct occupational categories (airline pilots, construction workers, and office employees). We hypothesized that these groups may exhibit differences in taste perception due to their unique occupational exposures and working conditions. Our primary objective was to examine taste identification time across the four basic tastes (sweet, salty, sour, and bitter) in these three groups [8]. By comparing taste perception across these diverse occupations, we aimed to elucidate any significant differences that may be attributed to specific work environments and exposures. Additionally, given the critical role of saliva in dissolving sensory stimuli and facilitating taste perception [22], we also compared salivary pH among the three study groups. By investigating these relationships, we aim to lay the groundwork for future research exploring the potential health implications of altered taste perception in different occupational settings and inform strategies for promoting sensory health and well-being among diverse working populations.

## Materials and methods

Study participants

This research forms part of an ongoing initiative to evaluate various health-related parameters across diverse occupational sectors [23-26]. The study employed a comparative methodology, focusing on three distinct professional groups: aviation personnel, construction industry workers, and office-based employees. Each cohort consisted of 30 male participants, who were recruited using convenience sampling methods from May to December 2023. Female participants were excluded due to their limited representation in the aviation sector [23-26]. To mitigate potential confounding factors, participants within each occupational category were matched for age and professional experience, with a minimum tenure requirement of five years. The study included only individuals in apparent good physical health, and smokers were not eligible to minimize the impact of this variable on the results. Exclusion criteria encompassed a history of systemic illnesses, alcohol consumption, cranial injuries, upper respiratory infections, or current medication regimens. This selection process aimed to ensure that any observed variations in gustatory perception could be primarily attributed to occupation-specific factors. Participant recruitment was facilitated by an occupational health specialist during routine health assessments conducted at outpatient facilities (Studio Minoretti, Oggiono, Italy). The research protocol adhered to the ethical principles outlined in the Declaration of Helsinki, with approval obtained from the relevant ethics committee (Studio Minoretti; reference number: 2022/GU). All participants provided written informed consent prior to their involvement in the study.

Measurements of salivary pH and taste identification time

The investigation was conducted in the morning, specifically between 09:00 and 10:00, to minimize potential bias arising from circadian rhythm variations. As an additional precautionary measure, participants were instructed to abstain from food, beverages, and tooth brushing for a minimum of two hours prior to the test. Following a thorough rinse of the oral cavity with tap water, salivary pH was measured immediately before recording the taste identification time. Unstimulated whole saliva samples (2 mL) were collected from each participant in sterile, disposable containers. The pH of each sample was measured using pH meter papers, with an accuracy of 0.25 units. Reference values for salivary pH typically range between 6.5 and 7.5 [27]. The subsequent taste identification time experiment utilized four distinct aqueous solutions, in accordance with previously established methodology [8]: a 40% sucrose solution for sweet taste, a 1% saline solution for salty taste, a 4% vinegar solution for sour taste, and a 20% unsweetened coffee solution for bitter taste. These aqueous solutions were administered to the study subjects using dissolvable strips of uniform size and shape. The taste strips were placed on the anterior two-thirds of the dorsal surface of the tongue. Upon application, a research assistant recorded the taste recognition time in seconds for each subject using a digital stopwatch.

Data analysis

To assess the normality of continuous variables, the Shapiro-Wilk test was used. The results demonstrated that all variables conformed to a normal distribution, thereby warranting the application of parametric statistical methods. Continuous data are presented as mean ± standard deviation, whereas categorical data are expressed as frequencies and percentages. Comparisons of continuous variables across the three study groups were conducted using one-way analysis of variance (ANOVA), with subsequent post hoc analysis employing Tukey's honest significant difference test. The chi-square test was used for the analysis of categorical variables. For correlation analyses, Pearson's product-moment correlation coefficient was calculated. All statistical analyses were performed using IBM SPSS Statistics for Windows, Version 20.0 (Released 2011; IBM Corp., Armonk, NY, USA). A two-tailed P-value <0.05 was considered statistically significant.

## Results

Table 1 provides an overview of the general characteristics of the three study groups. No significant intergroup differences were observed in terms of age, tenure of service, body mass index, total cholesterol, and fasting plasma glucose levels.

**Table 1:** General characteristics of the three study groups. ANOVA: analysis of variance; NS: not significant. ^*^Chi-square test. ^#^One-way ANOVA.

Variable	Airline pilots (n = 30)	Construction workers (n = 30)	Office employees (n = 30)	P-value
Men, n (%)	30 (100)	30 (100)	30 (100)	NS^*^
Age (years)	40.1 ± 3.5	39.7 ± 3.6	40.2 ± 3.7	NS^#^
Length of service (years)	10.5 ± 4.3	10.4 ± 4.5	10.6 ± 4.4	NS^#^
Body mass index (kg/m^2^)	24.5 ± 2.6	24.6 ± 2.5	24.6 ± 2.4	NS^#^
Total cholesterol (mg/dL)	214 ± 16	210 ± 19	218 ± 17	NS^#^
Fasting plasma glucose (mg/dL)	92 ± 11	94 ± 13	93 ± 12	NS^#^

The results concerning salivary pH and taste identification time are summarized in Table 2.

**Table 2:** Salivary pH and taste identification time in the three study groups. ANOVA: analysis of variance; NS: not significant. ^#^One-way ANOVA.

Variable	Airline pilots (n = 30)	Construction workers (n = 30)	Office employees (n = 30)	P-value
Salivary pH	7.25 ± 0.25	7.00 ± 0.50	7.25 ± 0.50	NS^#^
Sweet identification time, seconds	9.8 ± 3.9	7.0 ± 3.1	7.1 ± 3.3	<0.01^#^
Salty identification time, seconds	6.5 ± 3.1	6.7 ± 3.4	6.8 ± 3.2	NS^#^
Sour identification time, seconds	4.2 ± 2.6	6.1 ± 2.9	4.6 ± 2.5	<0.01^#^
Bitter identification time, seconds	4.4 ± 2.1	4.2 ± 1.8	4.1 ± 2.2	NS^#^

**Figure 1:**
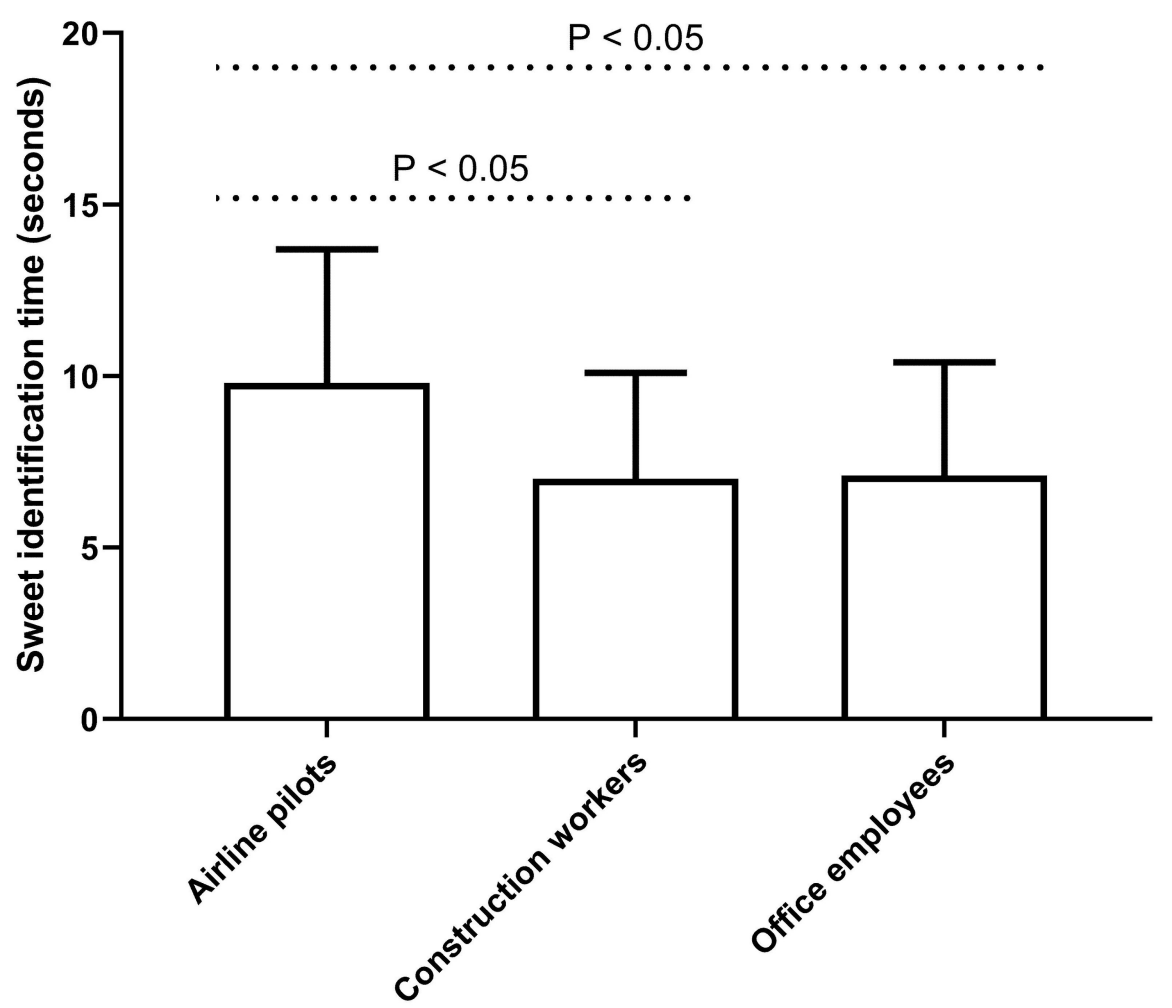
Sweet identification times in the three study groups. The P-values depicted in the figure were obtained through post hoc analyses using Tukey's honest significant difference test. This multiple comparison procedure was implemented to control for family-wise error rate and elucidate pairwise differences among group means following a significant one-way ANOVA test result. ANOVA: analysis of variance.

The analysis of salivary pH revealed no statistically significant differences among the three study groups. However, significant variations were observed in taste identification times, particularly for sweet and sour tastes (P < 0.01 for both, one-way ANOVA). As illustrated in Figure 1, airline pilots demonstrated a significantly prolonged sweet identification time (9.8 ± 3.9 seconds) compared to construction workers (7.0 ± 3.1 seconds, P < 0.05; post hoc Tukey's test) and office employees (7.1 ± 3.3 seconds, P < 0.05; post hoc Tukey's test), while no significant difference was found between the latter two groups.

**Figure 2:**
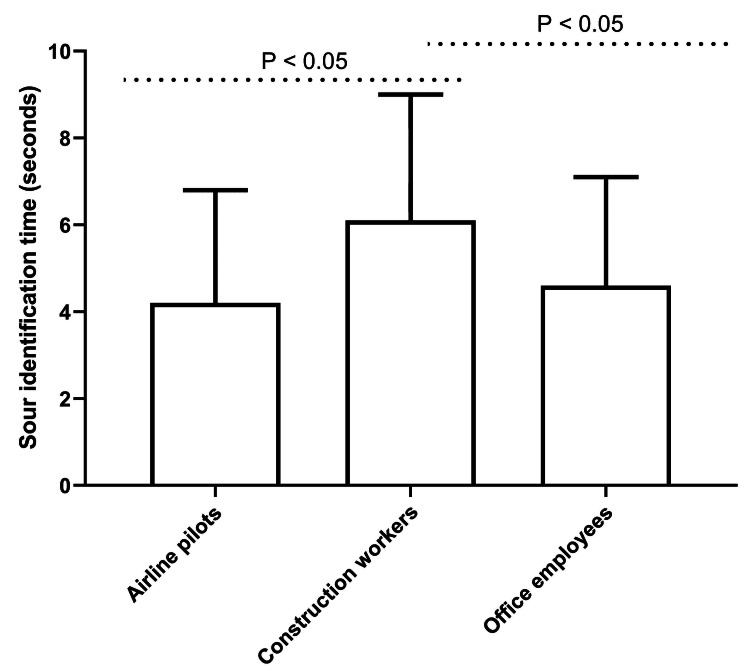
Sour identification times in the three study groups The P-values depicted in the figure were obtained through post hoc analyses using Tukey's honest significant difference test. This multiple comparison procedure was implemented to control for family-wise error rate and elucidate pairwise differences among group means following a significant one-way ANOVA test result. ANOVA: analysis of variance.

Conversely, construction workers exhibited a significantly extended sour identification time (6.1 ± 2.9 seconds) in comparison to both airline pilots (4.2 ± 2.6 seconds; P < 0.05; post hoc Tukey's test) and office employees (4.6 ± 2.5 seconds; P < 0.05; post hoc Tukey's test), as depicted in Figure 2. No significant differences were observed between airline pilots and office employees for sour taste identification. The identification times for salty and bitter tastes showed no significant variations among the three groups. Furthermore, salivary pH levels did not correlate with any taste identification time across the study groups (data not shown).

## Discussion

The results of this comparative study revealed intriguing differences in taste perception among airline pilots, construction workers, and office employees, particularly in the identification of sweet and sour tastes. These findings suggest that occupational factors may indeed influence gustatory function, albeit in a complex manner. However, no significant differences were observed in salivary pH among the three occupational groups. While this parameter plays a crucial role in taste perception by influencing the solubility and ionization of taste compounds [22,27], its uniformity across the groups suggests that any variations in taste perception are likely attributable to factors other than salivary composition.

The observed prolongation of sweet identification time in airline pilots compared to construction workers and office employees is a noteworthy finding that merits comment. This phenomenon may be attributed to the unique occupational stressors experienced by pilots, particularly frequent disruptions to their circadian rhythms and compromised sleep quality [16]. Previous research has established a connection between altered sleep patterns and changes in taste perception. Camargo et al. [28] demonstrated that individuals subjected to shifting work schedules or reduced sleep duration exhibit diminished sensitivity to sweet tastes compared to those maintaining regular sleep patterns and avoiding night work. This finding aligns with the results of Szczygiel et al. [12], who reported an increase in the preferred concentration of both sucrose and sucralose among individuals following a curtailed night of sleep compared to their habitual sleep duration. The extended sweet identification time observed in pilots may represent an adaptive response to their occupational challenges. This difference in taste perception could potentially serve as a compensatory mechanism to maintain energy homeostasis during irregular work schedules. The body's ability to modulate taste sensitivity in response to sleep disruptions and circadian misalignment may be an evolutionary adaptation to ensure adequate caloric intake under challenging conditions [12,13,28].

While we cannot rule out the possibility that factors such as fatigue, cognitive load, and environmental conditions in the cockpit may also contribute to the observed differences in sweet taste identification time, the potential long-term implications of a delayed sweet taste identification among pilots merit further investigation. Accordingly, the observed altered sweet taste perception may influence their dietary choices [21], potentially leading to increased sugar consumption to achieve the same level of sensory satisfaction. This could have long-term health consequences, particularly in relation to the potential onset of insulin resistance and metabolic disorders. Notably, airline pilots seem to exhibit a high prevalence of steatotic liver disease, which represents the hepatic manifestation of metabolic syndrome [29], as detected through transient elastography [30].

The observed extended sour taste identification time in construction workers presents a noteworthy observation that lacks an immediate explanation. One plausible hypothesis is that this prolonged response to sour stimuli may correlate with fatigue, a common condition in this occupational group. Previous research by Nakagawa et al. [31] indicates that engaging in physical tasks on an ergometer for 10-40 minutes significantly reduces the duration of sour aftertaste, suggesting a link between physical exertion and taste perception. Additionally, a Japanese study on female students found that higher fatigue scores were associated with diminished sour taste perception [32]. Sour taste is elicited by both organic and inorganic acids [33], and the receptor otopetrin-1, functioning as a zinc-sensitive proton channel, has been identified as a key component in this process [34]. While chronic exposure to airborne chemicals is known to compromise the integrity of taste receptor cells and surrounding tissues [14,15], it remains to be determined whether construction workers' exposure to various chemical agents and particulate matter may affect the function or expression of otopetrin-1. For construction workers, the prolonged sour taste identification time could lead to increased consumption of acidic foods, potentially impacting their overall dietary habits. Moreover, several electrolyte-rich beverages possess a slightly sour taste [35], and altered perception could influence hydration and electrolyte replenishment practices.

While this study provides pilot insights into occupation-related taste perception differences, several limitations should be acknowledged. The exclusion of female participants, necessitated by their underrepresentation in airline pilots, limits the generalizability of these findings. Considering that men and women differ in their taste sensitivities, with women being more sensitive to sweet and salty than men [36], future studies should strive to include a more diverse participant pool to account for potential sex-specific effects on taste perception. Additionally, the cross-sectional nature of this study precludes the establishment of causal relationships between occupational exposures and taste perception alterations. Longitudinal studies tracking taste perception changes over time in these occupational groups would provide more robust evidence of occupation-related effects. Investigations into taste receptor expression, neural plasticity, and the potential role of occupational stress hormones in modulating taste perception could offer valuable insights. Moreover, expanding the range of taste stimuli and incorporating umami taste perception would provide a more comprehensive understanding of occupation-related gustatory function [37]. Finally, future studies should explore the potential applications of taste modulation techniques in occupational settings where altered taste perception may influence dietary habits and health outcomes [38].

## Conclusions

This study reveals that occupational factors may significantly impact taste perception, particularly for sweet and sour tastes, among different professional groups. These insights emphasize the need to incorporate occupation-specific sensory changes into health and nutrition interventions tailored for diverse work environments. As research progresses, understanding the relationship between occupational exposures and sensory functions could lead to innovative strategies in occupational health and personalized nutrition. This evolving knowledge may ultimately enhance dietary recommendations and health management practices for various professions.
